# Translation, adaptation and validation of the American short form Patient Activation Measure (PAM13) in a Danish version

**DOI:** 10.1186/1471-2458-9-209

**Published:** 2009-06-29

**Authors:** Helle Terkildsen Maindal, Ineta Sokolowski, Peter Vedsted

**Affiliations:** 1 Department of General Practice, School of Public Health, Aarhus University, Aarhus, Denmark; 2 Research Unit for General Practice in Aarhus, Aarhus University, Aarhus, Denmark

## Abstract

**Background:**

The Patient Activation Measure (PAM) is a measure that assesses patient knowledge, skill, and confidence for self-management. This study validates the Danish translation of the 13-item Patient Activation Measure (PAM13) in a Danish population with dysglycaemia.

**Methods:**

358 people with screen-detected dysglycaemia participating in a primary care health education study responded to PAM13. The PAM13 was translated into Danish by a standardised forward-backward translation. Data quality was assessed by mean, median, item response, missing values, floor and ceiling effects, internal consistency (Cronbach's alpha and average inter-item correlation) and item-rest correlations. Scale properties were assessed by Rasch Rating Scale models.

**Results:**

The item response was high with a small number of missing values (0.8–4.2%). Floor effect was small (range 0.6–3.6%), but the ceiling effect was above 15% for all items (range 18.6–62.7%). The α-coefficient was 0.89 and the average inter-item correlation 0.38. The Danish version formed a unidimensional, probabilistic Guttman-like scale explaining 43.2% of the variance. We did however, find a different item sequence compared to the original scale.

**Conclusion:**

A Danish version of PAM13 with acceptable validity and reliability is now available. Further development should focus on single items, response categories in relation to ceiling effects and further validation of reproducibility and responsiveness.

## Background

Several initiatives have been taken to develop evidence-based activities to improve care for chronic conditions and, as in the Chronic Care Model, collaborative care and patient activation are cornerstones [1,2]. Evaluations of these initiatives are essential for further research and development. Although patient activation has been a central concept of chronic care for decades, there is a general lack of clarity regarding the definition of "activation", and consequently a lack of adequate assessment tools.

In 2004, Hibbard et al defined the concept of being "activated" and developed the Patient Activation Measure (PAM) 22 item and a 13 item short form (PAM13) [3,4]. They identified four elements; knowledge, skills, confidence and behaviours critical for coping with a chronic illness, and suggested four stages of activation that patients go through on their way to becoming fully activated in managing their own health [4,5]. Studies indicate that the PAM-measure predicts self-management behaviours, including healthy behaviours, disease-specific behaviours and "attitude to health system" – behaviour [6,7]. The PAM was formulated in two versions targeted people with or without chronic disease, with few semantic differences. Psychometric, PAM was evaluated to be a unidimensional, Guttman-like scale [3]. The PAM13 version for the chronically ill was used in this validation study (Table 1).

**Table 1:** Thirteen-Item Patient Activation Measure

**item**	
1	*When all is said and done, I am the person who is responsible for managing my health condition*
2	*Taking an active role in my own health care is the most important factor in determining my health and ability to function*
3	*I am confident that I can take actions that will help prevent or minimize some symptoms or problems associated with my health condition*
4	*I know what each of my prescribed medications do*
5	*I am confident that I can tell when I need to go get medical care and when I can handle a health problem myself*
6	*I am confident I can tell my health care provider concerns I have even when he or she does not ask*
7	*I am confident that I can follow through on medical treatments I need to do at home*
8	*I understand the nature and causes of my health condition(s)*
9	*I know the different medical treatment options available for my health condition*
10	*I have been able to maintain the lifestyle changes for my health that I have made*
11	*I know how to prevent further problems with my health condition*
12	*I am confident I can figure out solutions when new situations or problems arise with my health condition*
13	*I am confident that I can maintain lifestyle changes like diet and exercise even during times of stress*

The aims of this study were to translate and adapt the original American PAM13 into a Danish version and to report data quality and psychometric scale properties in a Danish population with dysglycaemia.

## Methods

### Translation and adaptation of the PAM13

A systematic approach to translation and adaptation was conducted as recommended by WHO [8]. It implies five steps: forward-translation, expert panel discussion, backward translation, a pre-test, a cognitive briefing and a consensus on the final version. Two independent native translators with Danish as their mother-tongue translated the original version of PAM13 from American English to Danish. These two translators comprised the expert panel together with three experts in chronic care and measurement development. The panel reconciled the forward translation into a single translation by identifying and resolving inadequate expressions or concepts. Back-translation was done as a quality control step to ensure that the original meaning of the concepts was derived. The back-translations were conducted by two independent native translators with American/English as their mother tongue and without knowledge of the questionnaire beforehand.

A pretest investigated the level of comprehensibility and cognitive equivalence of the translation [9,10] among 12 patients with newly diagnosed type 2 diabetes from a local diabetes outpatient clinic. The patients filled in the questionnaire at home and participated in a focus group interview on the following day. The interviewer (first author) facilitated a cognitive briefing on general comprehensiveness followed by a review of each question. The participants were asked to think out loud, highlight problems and express their attitude to the question. The final version of the questionnaire was resolved by the expert panel, and a simple and acceptable language was ensured in accordance with the WHO guidelines [8].

### Participants

The Danish version of PAM13 was sent to 467 participants in "The Ready to Act" health education randomised controlled study (296 in the intervention and 171 in the control group) at the 12-month follow-up [11]. Participants were between 43 and 75 years and diagnosed with different aspects of dysglycaemia (Impaired Fasting Glucose, Impaired Glucose Tolerance and Type 2 diabetes) by a step-wise screening procedure in general practice within the last five years [12,13]. Participants received a mail-administered PAM13 as part of a larger 16-page 1-year follow questionnaire on the psychological and behavioural outcomes of the "Ready to Act" study. A reminder including a new questionnaire was sent to participants who did not respond within three weeks.

### Ethics

Ethical approval of the study was attained from the local Science Ethics Committee of Aarhus County, Denmark (protocol no: 20000183). All participants gave informed content. The Danish Data Surveillance Authority permitted the collection and storing of data (journal no: 2000-41-0042).

### Scoring

Each item had five response categories with scores from 1 to 5: (1) strongly disagree, (2) disagree, (3) agree, (4) agree strongly and (5) not applicable. PAM is scored as a sum-scale. Only PAM-questionnaires with answers to seven or more items were included in the analyses. If included questionnaires had missing observations, these observations were omitted from the analysis, but not the corresponding persons or items. The raw scores were transformed into natural logarithms to achieve a better expression of the relative distances between the scores [14]. Further, items were calibrated from the logit metric to a user-friendly 0 to100 metric (0 = lowest activation level, 100 = highest activation) [15] to compare the Danish results to the original data.

### Analyses and statistical methods

The psychometric elements of the PAM13 Danish version were examined in two parts.

First, we assessed the data quality, internal consistency and correlations between items and the sum of the other items. Data quality was assessed in terms of mean for each item with standard deviation, median, percentage of missing data, number of "not applicable" answers and extent of ceiling and floor effects. Floor and ceiling effects between 1–15% were defined as optimal [16]. Internal consistency was assessed using Cronbach's alpha and average inter-item correlation. We defined an alpha of 0.80 as the lowest acceptable value [17-19]. In contrast to alpha, the average inter-item correlation is independent of the number of items and sample size when measuring internal consistency. We aimed at an average inter-item correlation between 0.15–0.50 [19].

We assessed whether each item had a high correlation with the sum score of the rest of the scale (internal item convergence), which is assumed in a unidimensional scale [17]. Correlations were fixed at a minimum of 0.60 to reflect a high level of internal convergence [20].

Secondly, we used Rasch Rating Scale Model [21,22] to investigate whether the scale was unidimensional, which is a prerequisite for the summation of the items [23]. The following criteria for Rasch model were investigated; item statistics, person and item reliability, rating scale diagnostics, factorial test of residuals and differential item functioning.

In the Rasch analysis, person and item scores were used to calibrate items on a logit scale where the midpoint of the scale is 0. Items at one end of the scale are "easier"/"less severe" and items at the other end are more "difficult"/"more severe". In the current analysis, items with a positive calibration were those indicating a high level of patient activation (more difficult to achieve).

From the Rasch model, we reported reliability and separation index for persons and items, and item statistics for measure order. Reliability expresses the reproducibility of the relative measure. A high reliability indicates that in all probability, persons (or items) with high measures actually do have higher measures than persons (or items) estimated with low measures. Winsteps [24] computes upper (Model) and lower (Real) boundary values for reliability. The true reliability can be found between these boundaries. Person reliability of 0.9 means that the scale may discriminate the sample into 3–4 levels, 0.8 into 2–3 levels and 0.5 into 1–2 levels. High item reliability merely indicates that the sample is big enough to precisely locate the items on the latent variable. We compared Rasch person reliability for subgroups to the original data [4].

An important characteristic of a high-quality scale is a good overall separation of persons and items assessed with the scale. The separation index is an estimate of the spread or separation of persons (or items) on the measured dimension. The separation index should be at least 2, indicating that the measure separated persons, items or both into at least two distinct groups [14]. Individual items that are at least 0.15 logits apart represent individual strata [25]. Otherwise one item is not distinctly separate from the next.

Two item fit mean square (MNSQ) statistics (infit and outfit) were computed to check whether the items fitted the expected model. MNSQ determines how well each item contributes to defining a single underlying construct (unidimensionality). Infit is more sensitive to misfitting responses to items closest to the person's ability level, while outfit is more sensitive to misfitting items that are farther away. If the data fit the Rasch model, the fit statistics should be between 0.6 and 1.4 [26].

The assumption is that the use of response categories for each item reflects the way people answer the items that are close to each other. However, this is only true if the distances between each response category are similar. The step measure (Rasch-Andrich threshold) is a calibrated measure of the transition between response categories. The thresholds are expected to increase monotonically. If not, the response categories do not reflect a reasonable interval on the latent variable, and consequently indicate substantial problems with the category definitions. Thresholds should increase by at least 1.4 logits, to show distinction between categories, but not more than 5.0 logits, to avoid large gaps in the variable [14].

Local independence of items was tested using Principal Components Analysis (PCA) on the Rasch item measure residuals. The purpose of PCA of residuals is to analyse the amount of unexplained variance and whether this unexplained variance indicates that there may be more than one dimension. Simulation studies indicate that even Rasch-conforming data produces residual-factors with eigenvalues up to 2.0. Thus, if there is more than one contrast (factors) in the residuals, there may be a second dimension. Contrasts in the Rasch analysis of residuals contradict unidimensionality [24].

As the last part of the Rasch analysis, we assessed differential item functioning (DIF) by estimating item parameters separately by groups of participants (sex, age groups, diagnosis, education, self-rated health and randomisation group). The scale should work uniformly, irrespective of the group assessed. The criteria used for the DIF analysis was DIF contrast >0.50. We tested using t-test and compared the probability multiplied by the number of DIF tests for each variable with a significance level of 0.05 to correct for multiple comparisons by the Bonferroni method.

Analyses were performed with Stata 10 [27] and Winsteps Rasch models software application [24].

## Results

### Translation and adaptation

The two translations from American to Danish agreed on most items. Different Danish words were used, but were semantically equivalent. A few conceptual discrepancies were identified; for example "health care", "medical care" and "treatment" had slightly different meanings, when used directly translated into Danish (item 5, 7, 9). The two translated versions were reconciled into a single translation relevant for Danish terminology at expert panel meetings between the translators and the research group.

The first back-translation included all items; the second back-translation included four items (item 2, 4, 7, 13). The emphasis in the back translation was on the conceptual and cultural equivalence, and not the linguistic as suggested by WHO [8]. We recognised a few general problems when comparing the two back-translations with the original version [4] and the first Danish draft: As in the forward-translation, we had difficulty translating health service terminology, partly because of organisational differences, and partly because a lack of specific Danish words for health care, illness and disease.

In the pre-test, the participants found all thirteen items relevant for measuring activation. The participants found the introductory wordings easy to understand; in addition they considered the response-categories exhaustive and exclusive. The participants found that the word "treatment" directed their attention to medication rather than diet or exercise. As half of the participants did not receive medication, they suggested that this elaborated term; "treatment (e.g. medicine, diet and exercise)" was used in the introductory wordings and in item two and seven. The expert group incorporated the results from the briefing process in the draft version, and proofreaders corrected the spelling and grammar.

### Participants

A total of 358 of 467 (76.7%) returned the questionnaire. Excluding "not applicable" answers, 344 had answered at least 50% of the items (>6 items) in PAM13. The mean age of the participants was 62.3 (s.d.: 7.1), 44.8% were female, 60.5% had type 2 diabetes and 39.5% had a pre-diabetic condition. The respondents had been diagnosed within the last five years (median 2 year (interquartiles 0–4).

### Data quality

The item response was high with few missing answers (0.8–4.2%) (Table 2). The response category "not applicable" was used by 0.6–18.4% of responders. In five of the items (4, 9, 11, 12 and 13) this category represented more than 10% of the answers. For all items, the distribution of answers was left-skewed with a small floor effect (range 0.6–3.6%) and a ceiling effect larger than 15% (range 18.6–62.7%) for all items. Cronbach's alpha was 0.89 and average inter-item correlation 0.38. Item-rest correlations (Table 2) ranged from 0.48–0.65 and were below 0.60 for six items (1, 4, 5, 6, 11 and 13).

**Table 2:** Data quality and item-rest correlations of the 13-items Patient Activation Measure Danish version in a population with dysglycaemia (n = 358).

**Item**	**N**	**Mean**	**SD**	**Median**	**Missing values**	**"Not applicable"**	**Floor**	**Ceiling**	**Item-rest correlation**
	**358**				**% of N = 358**	**% of N = 358**	**%**	**%**	
1	351	3.61	0.53	4	0.8	1.1	0.6	62.7	0.53
2	352	3.50	0.56	4	1.1	0.6	0.6	52.6	0.60
3	348	3.51	0.59	4	1.7	1.1	1.2	54.6	0.61
4	277	3.19	0.67	3	4.2	18.4	2.5	31.4	0.53
5	316	3.04	0.73	3	2.0	9.8	2.9	26.3	0.52
6	334	3.29	0.65	3	1.7	5.0	1.5	38.3	0.48
7	340	3.39	0.55	3	2.0	3.1	0.6	42.1	0.62
8	321	3.31	0.60	3	1.7	8.7	0.9	37.4	0.61
9	287	3.07	0.69	3	2.0	17.9	2.1	25.4	0.64
10	327	3.05	0.69	3	2.0	6.7	1.8	24.5	0.60
11	313	3.13	0.59	3	2.2	10.3	1.0	23.6	0.57
12	290	3.00	0.63	3	1.7	17.3	1.0	18.6	0.65
13	308	2.89	0.77	3	1.1	12.9	3.6	21.1	0.59

For the overall scale Cronbach's alpha was 0.89, average inter-item correlation 0.38

### Rasch analysis

#### Item statistics

The item infit and outfit mean square statistics ranged from 0.67–1.34, which all are within the acceptable range (Table 3). Separation distances of at least 0.15 logits were identified for nine of the 12 separations between items, but not for separations between items 2 and 3, items 10 and 9 and items 9 and 5 (Table 3). The calibrated 0–100 scale covered the range from 33.3–57.5.

**Table 3:** Thirteen-items Patient Activation Measure with Item Calibrations ordered by difficulty calibration

**Item**	**N = 344**	**Measure (logits)**	**SEM**	**Measure (0–100 scale)**	**SEM**	**Infit MNSQ**	**Outfit MnSQ**
1	342	-1.95	0.13	33.3	0.9	0.97	0.93
3	339	-1.38	0.12	37.3	0.9	0.98	0.94
2	342	-1.32	0.12	37.7	0.9	0.94	0.91
7	334	-0.76	0.12	41.7	0.9	0.82	0.81
8	319	-0.35	0.12	44.7	0.9	0.92	0.88
6	328	-0.21	0.12	45.7	0.9	1.34	1.30
4	277	0.30	0.13	49.3	0.9	1.23	1.20
11	313	0.53	0.12	50.9	0.8	0.86	0.87
10	324	0.85	0.11	53.2	0.8	0.99	1.01
9	287	0.86	0.12	53.3	0.8	0.91	0.94
5	314	0.86	0.11	53.3	0.8	1.28	1.29
12	289	1.11	0.11	55.1	0.8	0.67	0.69
13	305	1.45	0.11	57.5	0.8	1.05	1.16

**Measure (logits): **The estimate for the item difficulty in logits.

**Measure (0–100 scale): **The rescaled estimate for the item difficulty.

**SEM: **The standard error of measurement in estimation of the item difficulty. SEM is the precision of the item difficulty estimation and is shown in logits and 0–100 units.

**Infit MNSQ: **Infit mean square error is one of two quality control fit statistics assessing item dimensionality (the degree to which the item falls on the same single, real number line as the rest of the items). Infit is an information-weighted residual of observed responses from model expected responses and is most sensitive to item fit when the item is located near the person's scale location.

**Outfit MNSQ: **Outfit mean square error fit statistic is most sensitive to item dimensionality when the item scale location is distant from the person's scale location [4].

#### Person and item reliability

The overall Rasch person reliability for the Danish 13-item measure was between 0.83 (real) and 0.85 (model). Item reliability was 0.99. The separation index for persons was 2.24 and for items 8.37. Table 4 shows the person reliability statistics for subgroups in Danish populations compared with the American data. The person reliability was between 0.54 (real) and 0.92 (model). Some subgroups had a reliability below 0.80 (excellent self-rated health and age group 75 years or above).

**Table 4:** Reliability of 13-item Patient Activation Measure of the Danish version compared with the American version [4]

					**Rasch Person Reliability**			
			**PAM 13**	**PAM 13**	**Danish**		**American**	
	**N**	**%**	**Danish**	**American**	**Real**	**Model**	**Real**	**Model**
Sample	344	100	64.2	61.9	0.83	0.85	0.81	0.85
Gender								
Male	190	55.2	63.8	60.2	0.82	0.85	0.80	0.84
Female	154	44.8	64.7	62.8	0.85	0.86	0.82	0.85
Age group								
-54	52	15.1	62.5	63.9*	0.82	0.86	0.88*	0.91*
55–64	149	43.3	63.9	61.7	0.85	0.86	0.88	0.91
65–74	137	39.8	65.6	61.9	0.82	0.84	0.89	0.91
75+	6	1.7	56.6	58.2*	0.54	0.56	0.87*	0.90*
Self-rated health								
Poor	6	1.8	57.4	54.3	0.91	0.92	0.73	0.78
Fair	44	13.1	62.5	57.3	0.81	0.85	0.78	0.83
Good	189	56.4	62.6	59.3	0.82	0.85	0.78	0.82
Very good	83	24.8	66.6	64.3	0.84	0.86	0.79	0.83
Excellent	13	3.9	76.3	68.7	0.77	0.78	0.83	0.85
Education								
Unskilled	98	32.3	65.0	58.5^	0.82	0.84	0.75^	0.80^
Short (1–3 years)	130	42.9	62.8	61.8^	0.83	0.85	0.82^	0.86^
Higher (>3 years)	75	24.8	65.4	61.6^	0.82	0.85	0.82^	0.85^
Diagnosis								
Pre-diabetes	136	39.5	65.2	-	0.83	0.85	-	-
Type 2 diabetes	208	60.5	63.6	59.7	0.84	0.86	0.79	0.83

Only questionnaires with at least 7 items answered were included

* The extreme age group was 45–54 and 75–84 in the American version

^ Education in the American version is slightly different categorised: high school or less, some college and college graduate+

#### Rating scale diagnostics

The summary of measured steps is displayed in Table 5. This table shows the category label, observed counts, average measures, infit and outfit MNSQ, and step measures on the PAM13 scale. The category "Strongly disagree" was used in 1% of all answers whereas "agree" was used in 58% of all answers. However, both the average measure and the thresholds increased monotonically across the rating scale. The increase of the thresholds ranged from 1.4–4.2, which was within the targeted range.

**Table 5:** Response category measures and fit for 13-items Patient Activation Measure (n = 344)

**Response category**	**Observed count**	**%**	**Observed measure**	**Expected measure**	**Infit MNSQ**	**Outfit MNSQ**	**Treshold**
Strongly disagree	51	1	-0.30	-0.80	1.26	1.52	NONE
Disagree	368	10	0.18	0.17	1.03	1.03	-2.31
Agree	2185	58	1.59	1.63	0.91	0.88	-0.93
Agree strongly	1153	31	3.70	3.65	0.96	0.96	3.25

**Observed measure: **the average of the measures that are model led to produce the responses observed in the category

**Expected measure: **the expected value of the average measure for this sample

**Infit: **information-weighted fit statistic

**Outfit: **outlier-sensitive fit statistic

**MNSQ: **mean-square statistic with expectation 1.0

**Treshold: **the calibrated measure of the transition from the category below to this category. The bottom category has no prior transition, and so that the measure

#### Factorial test of residuals

PCA of item measure residuals revealed one dimension. A total of 43.2% of the variance in the data was explained by the measures and with a perfect model fit, this was expected to be 43.1%. The eigenvalue of the first PCA contrast was 2.5, which corresponded to 11% of the variance in the data.

#### Differential item functioning

No significant DIF was found in subgroups of self-rated health, diagnosis or randomisation groups. Items 1 and 2 were easier to endorse for highly educated persons compared to persons with short education (p-values Bonferroni-corrected) (DIF contrast = 1.35, *p *= 0.027 and DIF contrast = 1.22, *p *= 0.031). Item 10 was easier to endorse for men (DIF contrast = -0.65, *p *= 0.049) and item 13 was easier to endorse for persons between 65 and 74 years compared to persons under 55 (DIF contrast = 1.3, *p*<0.001) and compared to persons between 55 and 64 years (DIF contrast = 0.9, *p *= 0.023).

## Discussion

We found it possible to make a standardised translation and adaption of the original PAM13. The forward-backward translation was successfully conducted and the few discovered conceptual differences were primarily due to differences in health care systems. These findings are supported by the fact that e.g. the reliabilities in subgroups were comparable with the American version.

The psychometric assessment of the Danish version replicated to a great extent the findings from the original version [4] showing similar data quality and internal consistency. We found that the items had a different order. The items are arranged progressively in order of difficulty to reflect the developmental continuum of patient activation in an American population with chronic diseases. However, this was not confirmed by the results in this study, meaning that this population simply found some questions easier to answer compared with an American population. This could be due to the specific population of people with screen-detected newly diagnosed dysglycaemia compared with people with more manifest chronic diseases. If a similar item sequence is found in future studies in a Danish population, the PAM13 may need revision. The test items should be reordered to represent consecutive item difficulty, if the originally proposed model with four stages of activation identified by Hibbard et al [5] is to be of significance in the Danish version. Further, the person reliability did not indicate an ability to separate four levels at all. This may be due to differences between the Danish and the American populations, but as mentioned, the psychometric results in many instances replicate the American findings.

The investigation of the scale properties in general showed that PAM13 may be regarded as a unidimensional scale performing as a Guttmann sum-scale. This was particularly true for the reliability measures, measures of unidimensionality and aspects of the response categories. We found e.g. that the scale could distinguish between 2–3 levels as the person reliability was between 0.8–0.9, and the statistics indicated that each item could be regarded as part of one dimension (infit and outfit). The high-reliability estimates at the person level indicate that the scale is appropriate on an individual basis to diagnose activation and individualise plans for future health care as suggested [5].

However, we noted some possible problems with ceiling effect, potentially irrelevant items, and important aspects for responsiveness and separation difficulties at three points in the scale. Most PAM13 items demonstrated a ceiling effect and Items 1 to 3 had more than 50% of the answers in "agree strongly". This percentage suggests that the response categories do not cover relevant answers for the study population. Caution must be taken in future studies if ceiling effects are common in Danish populations. The high ceiling effect may be a problem if PAM13 is to be used for measuring change over time (e.g. in randomised studies) because of low responsiveness.

The five items with more than 10% answers in "not applicable" indicate that the scale cannot be used for all types of patients with chronic diseases and a revision of these items might be necessary.

On three points, there seemed to be no additional information when answering the next item in the scale (no separation). This means that 2–3 items can be omitted from the scale as a simple sum-scale. Further research will clarify the items to be omitted.

Although we may conclude that the Rasch analysis supports the PAM13 as a unidimensional sum-scale, some results do, however, indicate a need for improvement. We noted that six items had low correlations with the sum of the rest of the items, which indicates that they may not be absolutely true to one dimension. In addition, the test for other dimensions (PCA) revealed a possible additional factor. However, criteria for deciding whether there are two or more dimensions and when a deviation becomes a dimension have yet to be established. To the best of our knowledge, the rule of thumb is [24] that variance explained by measures four times greater than the variance explained by the additional factor and the size of the components less than three, is good. Our analysis does therefore not indicate more than one dimension.

When testing for differential item-function, most items did not have DIF in subgroups. However, items revealing DIF (item 1, 2, 10 and 13) showed possible explanations for this. Items 1 and 2 may appeal to educated people, being responsible and taking action. Gender seemed to play a role for item 10, which was endorsed by men more often than women, setting less demanding goals for their lifestyle changes. Patients aged 65–74 who answered more convincingly to item 13 may be explained by more experience of maintaining lifestyle even during stress.

The activation score in the Danish version covered the range from 33.3–57.5, which is more than the range of 38.6–53.0 for the American data [4]. However, this may not be enough to be able to detect changes in underlying behaviour studies, and in particular clinically relevant changes, which subsequently have to be tested.

### Strengths and limitations

The systematic translation approach was a strength in this study. Translation has no best practice as yet [9,28] and in particular, the value of back-translation has recently been questioned [29]. In our study, the backward translation procedure contributed with new perspectives on the cultural differences in the health care concepts.

We obtained a high response rate with 74% answering more than half of the items. This may minimise the risk of selection bias. The sample size of 344 persons was sufficient for this validation study as a minimum of 300 respondents is recommended to replicate structural analyses [19]. The mean square statistics used in the Rasch analysis are moderately insensitive of sample size for polytomous data [30].

The fact that PAM13 was delivered as part of a larger questionnaire at the 12-month follow-up of a health education intervention study might have affected the actual score level, but it is unlikely to have changed the scale properties. The number of missing values may have been higher that at the baseline questionnaire due the respondents being fatigued by the questionnaire.

The rather heterogeneous group of patients may be regarded as a weakness in many instances. However, when assessing scale properties, the use of a population representing many levels of activation is an advantage. A population screen-detected with dysglycaemia represents merely one of a range of chronic conditions.

## Conclusion

A Danish version of PAM13 measuring the latent variable of patient activation in chronic care is now available, although further development is recommended before use in daily practice. The PAM13 questionnaire was translated and adapted into Danish in a sample with screen-detected dysglycaemia showing initial reasonably good validity and reliability.

The Danish version formed a unidimensional, Guttman-like scale. The order of the items differed compared with the American version and therefore the suggested four activation stages in the American version were not relevant. Our findings show that the Danish PAM13 has promising psychometric properties indicating that going on with further validation in other populations with chronic diseases is expedient. However, special attention to discrimination and responsiveness is required to be able to use the score as a screening tool for tailored interventions. These studies have to be carried out before we have a much requested fully evidence-based activation measure for use in Danish chronic care intervention studies and in daily practice.

## Abbreviations

DIF: Differential Item Functioning; MNSQ: Mean Square; PAM13: Patient Activation Measure 13-items; PCA: Principal Components Analysis.

## Competing interests

The authors declare that they have no competing interests.

## Authors' contributions

HTM has contributed with the idea and the design and the acquisition of data. Furthermore, she has performed the data processing and interpreted the data in cooperation with a statistician. Finally, she wrote the first draft of the paper and did the revision. IS participated in the design of the study and performed the statistical analysis. PV conceived of the study together with HTM, and participated in its design and coordination and helped to draft the manuscript. All authors read and approved the final manuscript.

## Pre-publication history

The pre-publication history for this paper can be accessed here:


